# Japanese Spotted Fever and Irreversible Renal Dysfunction during Immunosuppressive Therapy after a Living-Donor Kidney Transplant

**DOI:** 10.3390/tropicalmed7080175

**Published:** 2022-08-10

**Authors:** Makoto Kondo, Kohei Nishikawa, Shohei Iida, Takehisa Nakanishi, Koji Habe, Keiichi Yamanaka

**Affiliations:** 1 Department of Dermatology, Mie University Graduate School of Medicine, 2-174, Edobashi, Tsu 514-8507, Japan; kmcasters@yahoo.co.jp (S.I.); t-nakanishi@med.mie-u.ac.jp (T.N.); habe-k@clin.medic.mie-u.ac.jp (K.H.); yamake@clin.medic.mie-u.ac.jp (K.Y.); 2 Department of Urology, Mie University Graduate School of Medicine, 2-174, Edobashi, Tsu 514-8507, Japan; kouheini@med.mie-u.ac.jp

**Keywords:** living-donor kidney transplant, Japanese spotted fever, atypical skin rash, immunosuppressive drugs

## Abstract

Ten years ago, a 56-year-old woman with a history of IgA nephropathy who received a living-donor kidney transplant across ABO barriers was managed with immunosuppressive drugs. The kidney transplant donor was her father who had poor kidney function. The patient’s renal function was stable for 10 years. The patient visited our department with a complaint of skin rash, occurring 2 days after an onset of fever. Although a skin rash is atypical for Japanese spotted fever (JSF), we suspected JSF and started treatment with minocycline because we found a scar suggestive of an eschar. Furthermore, the blood test results were similar to those associated with JSF, and the patient lived in a JSF-endemic area. The patient’s symptoms improved after 1 week. She was diagnosed with JSF by serological tests against *Rickettsia japonica*. JSF usually does not cause any complications after recovery. However, the patient’s renal function did not completely recover. JSF can cause an atypical rash in patients taking excessive immunosuppressive drugs. Early treatment is required for patients with suspected JSF to prevent complications of renal dysfunction after receiving a living-donor kidney transplant.

## 1. Introduction

*Rickettsia japonica* is the causative pathogen of Japanese spotted fever (JSF), which is transmitted by tick bites. It is characterized by a high fever, diffuse erythema on the whole body, and an eschar that forms at 2–8 days after a tick bite [1,2]. The delayed treatment of JSF leads to multiple organ failure (MOF) and disseminated intravascular coagulation (DIC) [1,3,4], and deaths have been also reported [4,5]. The administration of minocycline can quickly ameliorate the disease [1], and appropriate treatment does not cause sequelae of organ damage [1,3]. There have been no reports of JSF infection in patients receiving immunosuppressive drugs after renal transplantation. The current case highlights the possibility of irreversible renal damage after a rickettsial infection in a patient receiving immunosuppressive drugs.

## 2. Case Report

A 56-year-old woman with a history of IgA nephropathy, who had undergone a living-donor kidney transplant across ABO barriers 10 years ago, is receiving immunosuppressive therapy (tacrolimus, 1500 mg/day; mycophenolate mofetil, 750 mg/day; methylprednisolone, 2 mg/day; and everolimus, 1 mg/day). She was repeatedly hospitalized for cytomegalovirus (CMV) infection and other viral and bacterial infections, but had never developed any sequelae of renal dysfunction.

The kidney transplant donor was the patient’s father who had diabetes and hypertension and poor kidney function. For 10 years, renal function test results of the recipient revealed that the estimated glomerular filtration rate (eGFR) and cystatin C values were approximately 30–40 mL/min/1.73 m^2^ (normal range: 60 mL/min/1.73 m^2^) and 1.2–1.5 mg/L (normal range: 0.51–0.82 mg/L). The BUN and Cre values were 20–30 mg/dL (normal range: 8.0–20.0 mg/dL) and 1.2–1.5 mg/dL (normal range: 0.49–0.79 mg/dL), respectively. The amount of urinary protein detected at any time was up to 15 mg/dL (normal range: <30 mg/dL).

**Figure 1:**
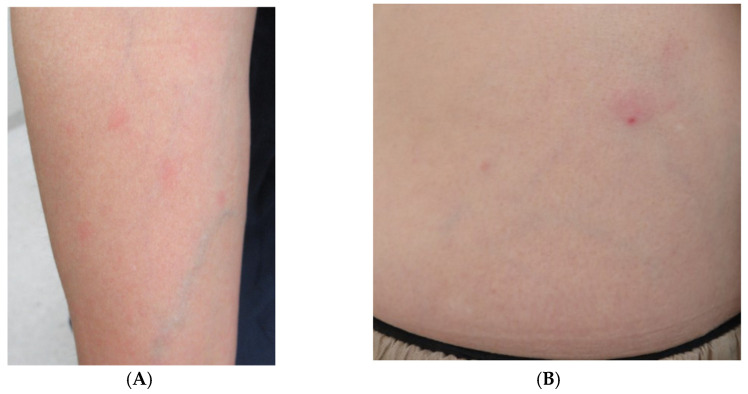
(**A**) Right arm with some circular erythematous macules without itching. (**B**) Erythema with a small black crust at the center noted on the patient’s abdomen.

One day, the patient presented to the emergency room complaining of fever and fatigue and was orally prescribed levofloxacin (500 mg/day). Then, the patient visited our department complaining of an appearance of skin rash without itching and persistent fever at 2 days after the onset of symptoms. Circular rashes on the arm and erythema with a small crust in the abdominal area were observed upon physical examination (Figure 1).

Laboratory findings reveal 528 mg/L (normal range: <14 mg/L) of C-reactive protein, 87 U/L (normal range: 7–23 U/L) of alanine aminotransferase, 107 U/L (normal range: 13–30 U/L) of aspartate aminotransferase, 331 U/L of LDH (normal range: 124–222 U/L), 32.6 mg/dL of BUN, 2.08 mg/dL of Cre, platelet count of 22.7 × 10^4^/μL (normal range: 15.8–34.8 × 10^4^/μL), and white blood cell count of 7870/μL (0% eosinophil count) (normal range: 3300–8600/μL, eosinophil count 0.6–8.3%). The renal function test results show an eGFR of 20.3 mL/min/1.73 m^2^ and a cystatin C value of 2.68 mg/L. The amount of urinary protein detected at any time was 100 mg/dL. The blood levels of mycophenolic acid, tacrolimus, and everolimus remained adequate. There were no findings suggestive of a worsening of the severe atrophy of the kidneys, and other abnormalities were not detected by computed tomography. A blood bacterial culture test had negative results. The CMV antigenemia test results using blood specimens were also negative. The skin biopsy of the erythema on the arm showed only a minimal perivascular infiltration of inflammatory cells. Although a skin rash is atypical for JSF, we started treatment with minocycline (100 mg/day for 1 week), because we found a scar on the patient’s abdomen suggestive of an eschar. Furthermore, the blood test results were similar to those associated with JSF, and the patient lived in a JSF-endemic area, specifically in the Ise-Shima area, which has the highest number of reported JSF cases in Japan. The patient had no travel history. As her JSF symptoms were mild, we decided to continue treatment for JSF on an outpatient basis. The fever and fatigue promptly resolved after treatment, and the skin rash had disappeared by the second visit (1 week after the first visit).

The polymerase chain reaction (PCR) assay of the blood and erythema specimens was negative for JSF. Subsequently, serological immunofluorescence tests against *R. japonica* were performed with the following results: acute-stage antibody (at first consult): immunoglobulin M (IgM) < 20 and IgG < 20 and recovery-stage antibody (3 weeks after the first consult): IgM < 20 and IgG 320. There are no reported cases of *Rickettsia* species other than *R. japonica* in the JSF-endemic area where the patient lives. Therefore, the patient was finally diagnosed with JSF. Her renal function did not recover completely: her eGFR was 25.8 mL/min/1.73 m^2^, cystatin C value was approximately 2.05 mg/L, BUN was 42.6 mg/dL, and Cre was 1.66 mg/dL, although we followed up on renal function for a year.

## 3. Discussion

JSF without the occurrence of DIC and/or MOF usually does not cause any complications after recovery [1]. The recovery of renal function is the usual outcome even in patients with Rickettsiosis, such as murine typhus and scrub typhus, with severe renal failure [6,7,8].

JSF is usually characterized by diffuse erythema throughout the body [1,2]. If a patient is taking immunosuppressive drugs at the time of an infection with a skin rash, the patient may not develop the typical skin rash that appears with that infection, because immunosuppressive drugs suppress cytokine production and release, causing vasodilation and collectively inhibiting the red blood cells and platelets in the blood. For this reason, the diagnosis of a rickettsial infection was difficult due to the characteristics of the skin rash though diffuse erythema with inflammatory cells infiltration forming on whole body in typical JSF. However, in the present case, we strongly suspected a rickettsial infection based on the patient’s laboratory data and place of residence. Therefore, we were able to intervene early by administering minocycline during the initial visit. It was also possible that the administration of levofloxacin prior to the appearance of the skin rash contributed to the mild symptoms of JSF experienced by our patient. There have been reports of improvement with levofloxacin in patients who were unable to receive minocycline [9].

The infectious disease usually causes more severe symptoms when patients are immunosuppressed. The detection rate of *R. japonica* gene by PCR assay of an erythema specimen is reported to be 80% [10]. *R. japonica* were detected in the areas of vasculitis; thus, the PCR assay of an erythema specimen showing vasculitis would likely show positive results [11]. Our patient had a negative result in the PCR assay of the erythema specimen for the detection of the *R. japonica* gene, indicating that fewer *R. japonica* were present. A previous report on the clinical manifestations of a kidney transplant patient infected with a tick-borne disease demonstrated that the symptoms were similar between transplant and non-transplant patients [12]. However, the patient underwent a living-donor kidney transplantation across ABO barriers and had to be more strongly immunosuppressed compared to the general kidney transplant recipients. Furthermore, JSF is more severely symptomatic compared to tsutsugamushi disease, which is caused by the overproduction of inflammatory cytokines [13]. The transplanted kidney was likely sub-optimal, as the donor had diabetes and hypertension, and this may have predisposed our recipient to damage caused by subsequent rickettsial infection, even if only few *R. japonica* were found. This may have resulted in subclinical infection by biting tick with a low number of *R. japonica* if a healthy person was bitten the same tick. It has been reported that subclinical infection is possible in rickettsial infections [14,15].

JSF results in an intense inflammatory cytokine storm [16]. Furthermore, direct damage to the kidneys due to rickettsial infections and decreased renal blood flow due to systemic vasculitis was reported [17,18,19]. Because *Rickettsia* invades the endothelial and smooth muscle cells, a histologic analysis reveals vasculitis with peritubular capillaries and venules [20]. As vascular cells become necrotic, macrophages and extravasated erythrocytes are found. Fibrin thrombosis may form in the renal vessels because the microcirculation is destroyed [21].

The patient was forced to undergo excessive immunosuppressive therapy after a living-donor kidney transplant across ABO barriers. Although mild JSF cases would not normally result in renal damage, the direct effects of inflammatory cytokines to the kidney, effect of renal vasculitis, reduced renal blood flow, excessive immunosuppression, and condition of donated kidney may have caused irreversible damage to the renal function of the transplanted kidney in patients on immunosuppressive therapy. A delayed diagnosis of JSF would have resulted in a more severe irreversible renal dysfunction. It should be kept in mind that skin rashes associated with infections can be atypical in immunosuppressed cases.

## 4. Conclusions

We presented a case of JSF in a renal transplant recipient maintained on high levels of immunosuppression in the late post-transplant period. Even in mild JSF cases, early treatment is required for patients with suspected JSF to prevent complications of renal dysfunction after a living-donor kidney transplant. We must keep in mind that when patients receive excessive immunosuppressive drugs, the skin rash resulting from that infection may not take its typical form.

## Data Availability

The generated and analyzed datasets will be available upon request to the corresponding author.
